# Genome Sequence of *Enterococcus pernyi*, a Pathogenic Bacterium for the Chinese Oak Silkworm, *Antheraea pernyi*

**DOI:** 10.1128/genomeA.01764-15

**Published:** 2016-05-19

**Authors:** Ying Sun, Xisheng Li, Guobao Wang, Yong Wang, Yiren Jiang, Yanqun Liu, Zhiguo Yu, Li Qin

**Affiliations:** a College of Plant Protection, Shenyang Agriculture University, Shenyang, China; b College of Bioscience and Biotechnology, Shenyang Agriculture University, Shenyang, China; c Sericultural Research Institute of Liaoning Province, Fengcheng, China

## Abstract

We report the draft genome assembly of *Enterococcus pernyi*. The genome sequence is 3.09 Mb in length with a G+C content of 38.35%. It covers 3,153 genes with an average length of 854 bp, and contains 65 tRNAs, 13 small RNAs, and 18 rRNAs. Moreover, it contains 9 genomic islands with an average length of 14,058 bp and 3 prophages with an average length of 37,430 bp.

## GENOME ANNOUNCEMENT

The Chinese oak silkworm (*Antheraea pernyi* [Guérin-Méneville, 1855]), which belongs to *Lepidoptera*: *Saturniidae*, is the most well-known wild silkworm for insect food and silk production. Rearing of the Chinese oak silkworm has a history of about 400 years in China (1). Empty-gut disease is one of the most important diseases in *A. pernyi*, and this disease seriously affects the yield of the tussah cocoon and causes great economic losses. The pathogen of the disease was established as *Streptococcus pernyi* sp. nov., based on morphological, physiological, biochemical, and serological characteristics (2). However, *S. pernyi* sp. nov. has been reclassified and renamed as *Enterococcus pernyi*, based on phylogenic analysis of 16S rRNA and *tuf* gene sequences (3–5). Now, the pathogen of empty-gut disease in *A. pernyi* has been defined as *E. pernyi* in the taxonomy of the National Center for Biotechnology Information. The genome of *E. pernyi* was sequenced to gain a better understanding of the taxonomic status of this bacterium and provide more genomic information for further studies to prevent and cure the disease.

The complete genome sequence was determined by Illumina Solexa technology at Novogene Bioinformatics Technology Co., Ltd. (Beijing, China). Sequence assembly was performed using SOAPdenovo version 2.04 (6). Coding sequences (CDSs) were predicted using GeneMarkS software (7) and further annotated into databases through BLASTp, including NCBInr, COG, GO, KEGG, Swiss-Prot, and TrEMBL. tRNAscan (8), RNAmmer (9), and Rfam (10) were used to predict tRNAs, rRNAs, and small RNAs, respectively. Gene islands and prophages were predicted using IslandPath-DIOMB (11) and PHAST software (12).

The genome size of *E. pernyi* is 3.09 Mb with a G+C content of 38.35%. A total of 626 Mb of clean data were generated, reaching a genome coverage depth of over 200-fold. Sequences were assembled into 23 contigs with a total length of 3,181,210 bp (largest, 603,828 bp, and smallest, 654 bp) and with an *N*_50_ contig size of 370,188 bp. Finally, there were a total of 9 scaffolds with a total length of 3,188,572 bp (largest, 3,086,269 bp, and smallest, 654 bp) and with an *N*_50_ scaffold size of 3,086,269 bp. The genome contains 3,153 CDSs with an average length of 854 bp, which represent 84.48% of the whole genome. The annotation results showed that only 224 CDSs (7.1%) were not annotated into any databases; there were 2,916, 1,537, 1,577, 1,487, 1,242, and 2,812 CDSs annotated into NCBInr, COG, GO, KEGG, Swiss-Prot, and TrEMBL, respectively. Meanwhile, 65 tRNAs, 18 rRNAs, and 13 small RNAs were identified. Furthermore, the genome contains 9 genomic islands with an average length of 14,058 bp, and contains 3 prophages with an average length of 37,430 bp. There was no clustered regularly interspaced short palindromic repeat identified in the genome.

### Nucleotide sequence accession number.

The whole-genome sequences of *E. pernyi* have been deposited at DDBJ/EMBL/GenBank under the accession number LPVT00000000.
